# Alterations in mitochondria-endoplasmic reticulum connectivity in human brain biopsies from idiopathic normal pressure hydrocephalus patients

**DOI:** 10.1186/s40478-018-0605-2

**Published:** 2018-10-01

**Authors:** Nuno Santos Leal, Giacomo Dentoni, Bernadette Schreiner, Olli-Pekka Kämäräinen, Nelli Partanen, Sanna-Kaisa Herukka, Anne M Koivisto, Mikko Hiltunen, Tuomas Rauramaa, Ville Leinonen, Maria Ankarcrona

**Affiliations:** 1 Center for Alzheimer Research, Division of Neurogeriatrics, Department of Neurobiology, Care Sciences and Society, Karolinska Institutet, Novum 5th floor, SE-141 57 Huddinge, Sweden; 2 Institute of Clinical Medicine – Neurosurgery, University of Eastern Finland, Kuopio, Finland; 3 Department of Neurosurgery, Kuopio University Hospital, Kuopio, Finland; 4 Institute of Clinical Medicine – Neurology, University of Eastern Finland, Kuopio, Finland; 5 Department of Neurology, Kuopio University Hospital, Kuopio, Finland; 6 Institute of Biomedicine, University of Eastern Finland, Kuopio, Finland; 7 Institute of Clinical Medicine – Pathology, University of Eastern Finland, Kuopio, Finland; 8 Department of Pathology, Kuopio University Hospital, Kuopio, Finland; 9 Unit of Clinical Neuroscience, Neurosurgery, University of Oulu, Oulu, Finland; 10 Medical Research Center, Oulu University Hospital, Oulu, Finland

**Keywords:** Brain biopsies, iNPH, Amyloid β-peptide, Tau, MERCS, MAM

## Abstract

Idiopathic normal pressure hydrocephalus (iNPH) is a neuropathology with unknown cause characterised by gait impairment, cognitive decline and ventriculomegaly. These patients often present comorbidity with Alzheimer’s disease (AD), including AD pathological hallmarks such as amyloid plaques mainly consisting of amyloid β-peptide and neurofibrillary tangles consisting of hyperphosphorylated tau protein. Even though some of the molecular mechanisms behind AD are well described, little is known about iNPH. Several studies have reported that mitochondria-endoplasmic reticulum contact sites (MERCS) regulate amyloid β-peptide metabolism and conversely that amyloid β-peptide can influence the number of MERCS. MERCS have also been shown to be dysregulated in several neurological pathologies including AD.

In this study we have used transmission electron microscopy and show, for the first time, several mitochondria contact sites including MERCS in human brain biopsies. These unique human brain samples were obtained during neurosurgery from 14 patients that suffer from iNPH. Three of these 14 patients presented comorbidities with other dementias: one patient with AD, one with AD and vascular dementia and one patient with Lewy body dementia. Furthermore, we report that the numbers of MERCS are increased in biopsies obtained from patients diagnosed with dementia. Moreover, the presence of both amyloid plaques and neurofibrillary tangles correlates with decreased contact length between endoplasmic reticulum and mitochondria, while amyloid plaques alone do not seem to affect endoplasmic reticulum-mitochondria apposition. Interestingly, we report a significant positive correlation between the number of MERCS and ventricular cerebrospinal fluid amyloid β-peptide levels, as well as with increasing age of iNPH patients.

**Electronic supplementary material:**

The online version of this article (10.1186/s40478-018-0605-2) contains supplementary material, which is available to authorized users.

## Introduction

Idiopathic normal pressure hydrocephalus (iNPH) is a neurological disease with unknown aetiology, characterised by gait and cognitive impairment as well as enlarged cerebral ventricles (ventriculomegaly) [37]. The exact molecular mechanisms underlying this pathology are still unknown. Currently, the only available treatment for iNPH, is the implementation of a surgical CSF shunt which alleviates the symptoms in certain patients [15]. Interestingly, iNPH patients often present comorbidity with Alzheimer’s disease (AD) [17]. AD is characterized by two major hallmarks: extracellular amyloid plaques, mainly consisting of amyloid β-peptide (Aβ), and intracellular neurofibrillary tangles (NFT), consisting of hyperphosphorylated tau (pTau) protein [29]. Due to the often co-existing lesions in these two diseases, it is important to understand the specific mechanistic differences and similarities between AD and iNPH to better diagnose and treat patients.

Recent studies from our and other laboratories have highlighted the role of mitochondria-endoplasmic reticulum (ER) contact sites (MERCS) in neurodegenerative disorders [1, 5, 18, 28, 33]. MERCS are formed where the outer mitochondrial membrane interacts with a specific sub-region of ER that presents a lipid raft-like domain commonly known as mitochondria associated membranes (MAM) [7]. MERCS are involved in several cellular mechanisms like Ca^2+^-shuttling from ER to mitochondria, phospholipid metabolism, autophagosome formation and Aβ metabolism [22]. Changes in MERCS have been shown in a variety of diseases like AD, cancer, diabetes, obesity, Parkinson’s disease, traumatic brain injury and FTD/ALS.

However, structural analysis of MERCS in human brain material has so far not been performed. In this study we, for the first time, have visualized MERCS and other types of mitochondria contacts in a unique material: brain biopsies from patients undergoing iNPH reversal surgery. Furthermore, as iNPH patients often have AD-related lesions, we saw this material as an opportunity to assess any potential connection of the pathological hallmarks seen in AD and contact formation. We analysed MERCS in iNPH sufferers by grouping patients based on their dementia diagnose or presence of amyloid plaques and NFT at time of surgery. Interestingly, we detected an increased number of MERCS per cell profile in samples from patients diagnosed with dementia (Lewy body dementia (LBD), vascular dementia (VaD), AD). Positive correlations between the number of MERCS per cell profile and age as well as ventricular cerebrospinal fluid (CSF) Aβ42 levels were also detected.

## Material and methods

### Human brain biopsies

Human brain biopsies were obtained as previously described [26]. In brief, a right frontal 12-mm burr hole was made three centimetres laterally from the midline close to the coronal suture in anesthetized iNPH patients. One to three cylindrical cortical biopsies (2-5 mm in diameter, 3-7 mm in length) were taken using disposable 14G biopsy needle (Temno™, BD, Franklin Lakes, NJ, USA). The biopsy material was placed in fixative solution (1% glutaraldehyde and 3.7% formaldehyde in sodium phosphate buffer) 10 min after collection. The samples were kept in fixative solution 4–14 days and subsequently embedded into the paraffin. Consecutive 7 μm thick sections were stained with hematoxylin-eosin (HE) and immunohistochemistry (IHC) including pTau (MN-1020, clone AT8 IGH 135; Thermofisher) and Aβ (6F/3D, M0872; Dako). Stained sections were assessed under light microscopy at × 100 to × 200 magnifications. Cellular or neuritic pTau-structures were identified and rated as negative or positive. In Aβ-IHC stained sections, fleecy, diffuse and dense plaques were assessed (Dr Rauramaa) and the staining results of Aβ semi quantitatively rated [31].

A total of 14 human biopsies from 14 patients were analysed (age range 71 to 86 years, average 77.3 years old, 28.6% males and 71.4% females). The same patients were stratified in two different ways: first in two groups according to presence or absence of dementia diagnose and second in three groups according to the presence of amyloid plaques and NFT (Aβ^+^/tau^+^), plaques only (Aβ^+^/tau^−^) or negative staining (Aβ^−^/tau^−^) (Table 1 and Additional file 1: Figure S1).

**Table 1:** Clinical data collected from iNPH patients

	#	Gender	Age	Comorbidities	MMSE	CSF (lumbar) in ng/L			CSF (ventricular) in ng/L		
						Aβ42	p-Tau	Total-Tau	Aβ42	p-Tau	Total-Tau
Aβ^−^/tau^−^	1	F	75	NI	22	NA	NA	NA	1286,98	93,14	827,59
	2	F	76	NI	23	655,03	20,88	90,38	655,03	31,56	316,31
	3	F	77	NI	25	904,05	35,99	185,81	311,68	28,50	228,98
	4	M	75	NI	19	1092,13	36,74	292,90	650,03	42,09	625,81
Aβ^+^/tau^−^	5	M	86	LBD/VaD	13	NA	NA	NA	NA	NA	NA
	6	F	79	NI	24	833,23	32,66	225,14	817,66	35,22	328,78
	7	M	79	NI	19	489,59	25,66	225,62	124,05	46,87	1001,38
	8	F	71	NI	20	785,34	24,49	128,72	618,75	123,54	3122,29
	9	F	76	NI	23	463,30	36,63	218,42	326,77	74,43	1118,52
Aβ^+^/tau^+^	10	F	78	NI	23	611,61	38,54	228,68	502,86	52,52	505,93
	11	F	77	NI	28	860,71	28,01	152,77	464,72	70,77	1619,43
	12	F	74	NI	24	876,30	57,34	311,77	281,83	46,13	506,74
	13	M	79	AD/VaD	14	436,77	29,93	176,40	282,76	111,23	2252,69
	14	F	81	AD	15	695,54	52,00	470,42	569,76	82,00	1005,13

*NA* Not available, *NI* Non-identified

### CSF sampling and analysis

CSF samples were obtained by lumbar puncture during diagnostic tap-test at outpatient clinic or during insertion of intraventricular catheter. Low protein binding PP tubes were used. Samples were centrifuged, divided into 1 mL tubes and frozen at − 80 °C. CSF AD biomarkers (total tau, pTau_181_, Aβ_1–42_) were measured at the University of Eastern Finland (UEF) Neurology using INNOTEST ELISA kits (Fujirebio Europe, Ghent, Belgium).

### MMSE

The Mini-Mental State Examination (MMSE, range 0–30) was used to evaluate patients’ cognitive function [8]. Patients were classified into three groups: no significant cognitive impairment (27 ≤ MMSE ≤30), minor cognitive impairment (23 ≤ MMSE ≤26) or moderate or severe cognitive impairment (MMSE ≤22) [16].

### Transmission electron microscopy (TEM) and image analysis

Ultrathin sections from human biopsies were processed using Leica Ultracut UCT (Leica, Vienna, Austria) and contrasted with uranyl acetate and lead citrate. Sections were observed with a Tecnai 12 BioTWIN transmission electron microscope (FEI Company, Eindhoven, The Netherlands) at 100 kV. Digital images were acquired with a Veleta camera (Olympus Soft imaging Solutions, GmbH, Münster, Germany) at a primary magnification of 20.500×.

Pictures were acquired as before [6]. Briefly, 10 random cells were chosen per patient and, for each cell, pictures of all visible mitochondria were taken. In total, 140 cells were analysed, including more than 800 MERCS and 2000 mitochondria. The number of MERCS and mitochondria as well as MERCS length and mitochondria perimeter were obtained using iTEM FEI software (EMSIS GmbH, Muenster, Germany). MERCS were considered as such when the distance between ER and mitochondria was equal or bellow 30 nm. Values presented in Additional file 1: Table S1 represent average values per cell profile per patient. The overall quality of the tissue was very high and representative images are shown. In particular, the structure of mitochondria was very well preserved while the ER structures sometimes appeared dilated.

### Statistical analysis

Data were analysed using IBM SPSS Statistics 24 software (IMB Corportation, New York, NY, USA). Data did not follow normal distribution and therefore samples were compared two by two by non-parametric independent test (Mann-Whitney *U* test). For correlation studies the Pearson correlation coefficient (r) was used as our data was numeric and continuous. All values are expressed as mean ± SEM, n = correspond to number of patients, * *p* < 0.05 was considered to be significant.

## Results

### Organelle contact sites in human brain biopsies

**Fig. 1:**
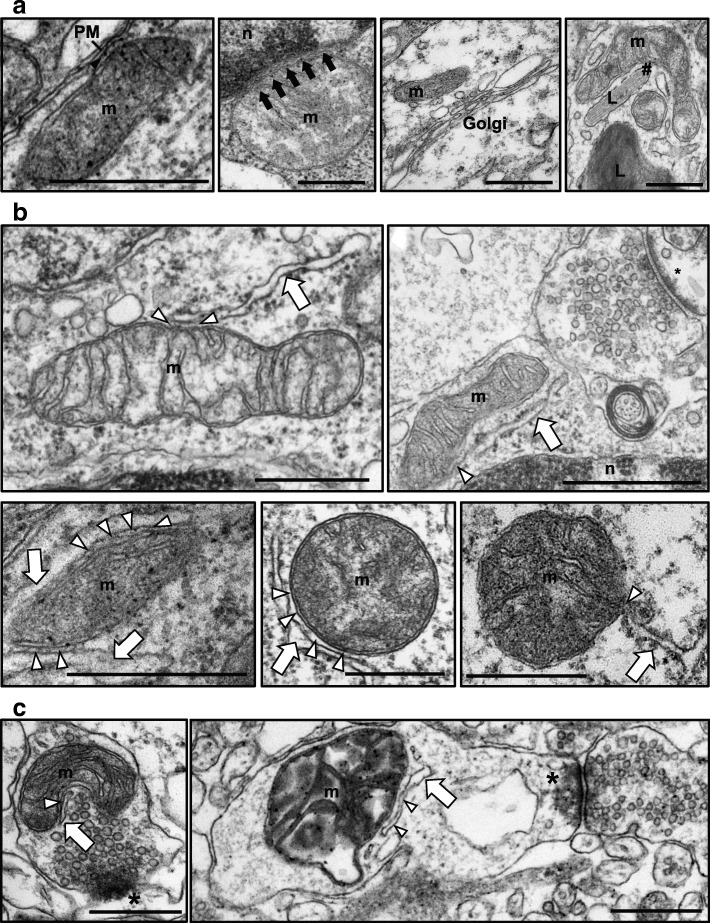
Selected electron micrographs of human brain biopsies from iNPH patients. **a** Interaction of mitochondria with plasma membrane (PM), nucleus (n), Golgi and lisosomes (L) (from left to right). **b** Interactions between ER and mitochondria (m). Small stretches of ER in contact with mitochondria (top left and right), longer contacts of ER in contact with mitochondria (bottom panel, left and middle), and a point contact (bottom, right). **c** MERCS in pre-synaptic (left) and post-synaptic (right) density. Black arrow head – mitochondria-PM interaction, black arrow – mitochondria-nucleus interaction, # – mitochondria-lysosome interaction, white arrow – ER, white arrow head – MERCS, * - synapse. Scale bar = 500 nm

Cellular organelles interact with each other through membrane contact sites [14]. Here we have identified such contacts using TEM in fixed human brain biopsies. Several diverse organelle contacts were identified including: mitochondria-plasma membrane (PM) (Fig. 1a, left) [36], mitochondria-nucleus (Fig. 1a, middle left) [24], mitochondria-Golgi (Fig. 1a, middle right) [4] and mitochondria-lysosome (Fig. 1a, right) [12]. However, the most common membrane contact site observed were MERCS [35] (Fig. 1b). In fact, 12.8 ± 0.5% of the mitochondria profile surface was found to be in contact with ER and, on average, 45.9 ± 3.4% of mitochondria were in contact with at least one stretch of ER. In accordance with previous studies [10], we also detected different types of MERCS in human brain in our TEM analysis. While in some electron micrographs only a part of ER is in contact with mitochondria (Fig. 1b, top panels), other show long extensions of interactions between the two organelles (Fig. 1b, bottom left and middle panel). There are also examples where just a branch of ER touches the outer mitochondrial membrane (Fig. 1b, bottom right panel). Moreover, as recently reported in mouse brain tissue, we also detected MERCS in the pre- and post-synaptic terminals in human brain (Fig. 1c, left and right, respectively) [38].

### Patients diagnosed with dementia and with lower MMSE showed increased number of MERCS

**Fig. 2:**
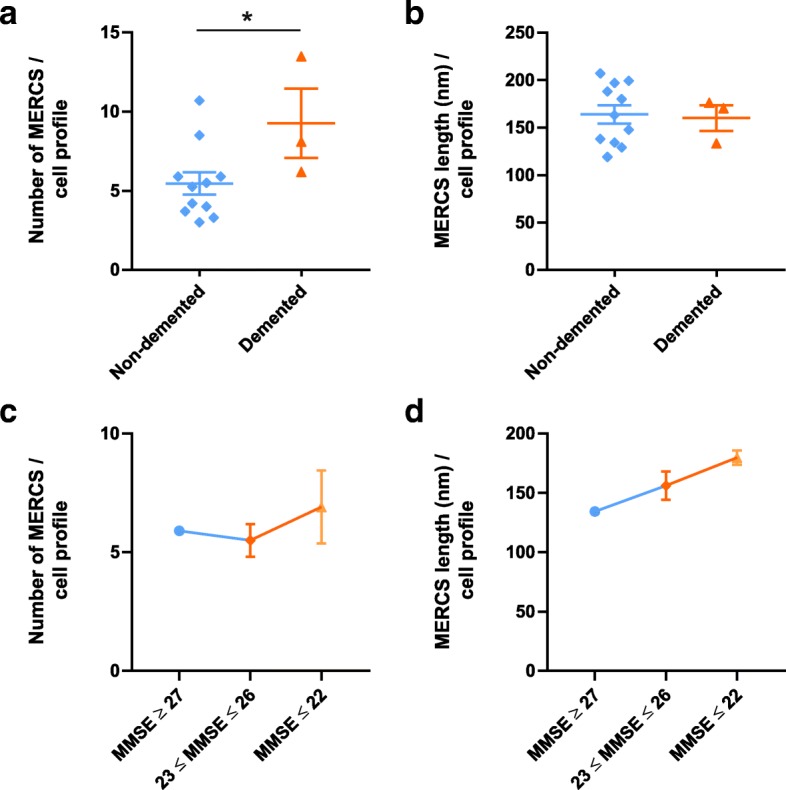
Patients diagnose with dementia and with lower MMSE present increased MERCS. Quantification of **a** number and **b** length of MERCS from the electron micrographs of iNPH patients’ biopsies according to dementia diagnose. Non-demented patients are #1 to #4 and #6 to #12, demented patients are #5, #13 and #14. Each point represent one iNPH patient. Quantification of **c** number and **d** length of MERCS from the electron micrographs of iNPH patients’ biopsies according to MMSE. MMSE scores represent: MMSE ≥27 – No significant cognitive impairment, 23 ≤ MMSE ≤26 – Minor cognitive impairment, MMSE ≤22 – moderate or severe cognitive impairment

Several studies suggest that MERCS are dysregulated in different neurological disorders [1, 13, 25, 33]. Here we aimed to study MERCS in human brain biopsies collected from iNPH patients. Therefore, we stratified the clinical data in different groups. Among the 14 patients analysed, only three were diagnosed with at least one type of specific dementia (#5 LBD/VaD, #13 VaD/AD, #14 AD). Interestingly, demented patients presented a higher number of MERCS per cell profile as compared to non-demented (Fig. 2a) while no differences were observed in MERCS length per cell profile (Fig. 2b). In addition, patients with moderate or severe cognitive impairment (MMSE ≤22) presented a higher number of MERCS per cell profile as well as MERCS perimeter when compared to patients with mild cognitive impairment (23 ≤ MMSE ≤26) or no significant cognitive impairment (27 ≤ MMSE ≤30) (Fig. 2c and d). Since only one patient presented MMSE ≥27 no statistical analysis was performed. To confirm these data, we performed correlation studies between MMSE and MERCS number and length (Additional file 1: Figure S3). For both cases we saw that there was a negative correlation between MMSE and number (Additional file 1: Figure S3a) and length (Additional file 1: Figure S3b) of MERCS. Since mitochondria surface area could influence MERCS we also measured number of mitochondria profile and perimeter. No significant differences were observed (Additional file 1: Figure S2a-d).

### Number of MERCS correlates with age and ventricular CSF Aβ42 levels

**Fig. 3:**
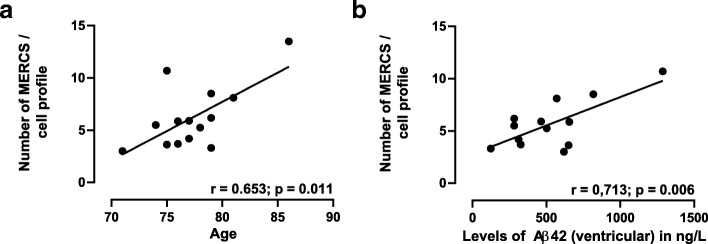
Number of contacts positively correlate with age and levels of Aβ42. Representation of the correlation between mitochondria-ER contacts **a** with age and **b** with ventricular levels of Aβ42. Linear regression was performed and the Pearson correlation coeficient (r) calculated. Each point represent one iNPH patient

As we had access to several clinical parameters collected from the iNPH patients (Table 1), we used Pearson’s correlation coefficient in order to identify possible correlations with the number and/or length of MERCS. We found that the number of MERCS had a significant positive correlation with increasing patients’ age (*r* = 0.653, *p* = 0.011) (Fig. 3a). Like mentioned before, iNPH patients often present comorbidity with AD. We found that there was a significant positive correlation between the number of MERCS and the levels of ventricular CSF Aβ42 (*r* = 0.713, *p* = 0.006) (Fig. 3b). Although, no significant correlations were detected between number of MERCS and lumbar CSF Aβ42 nor between MERCS, pTau and total tau levels.

### The presence of amyloid plaques and NFT correlates with shorter MERCS in human brain

**Fig. 4:**
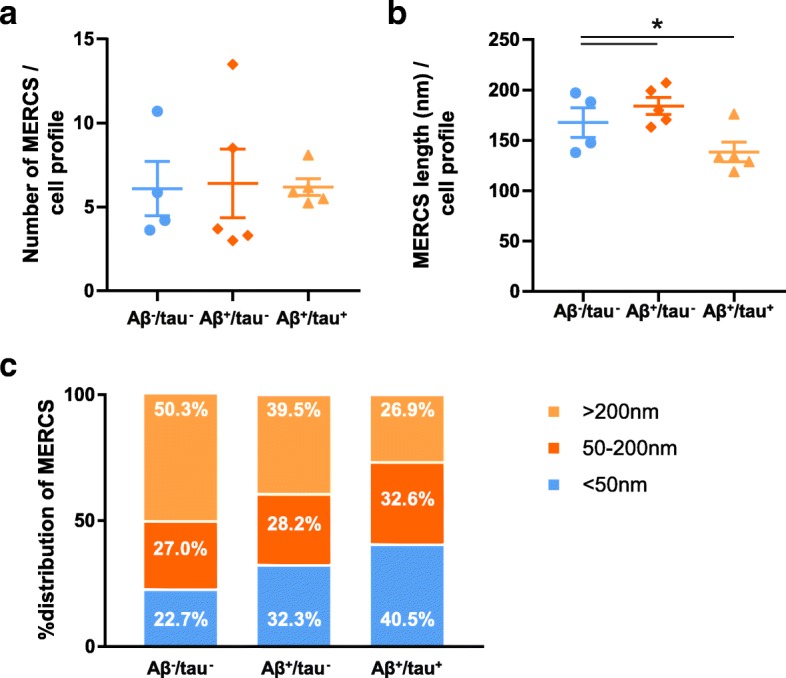
Patients with amyloid plaques and NFT present smaller contacts. Quantification of **a** number and **b** length of mitochondria-ER contacts from the electron micrographs of iNPH patients biopsies. **c** Representation of the % of distribution of contacts according to their length. Aβ^−^/tau^−^ patients present no amyloid plaques nor NFT; Aβ^+^/tau^−^ patients presents amyloid plaques but not NFT; and Aβ^+^/tau^+^ patients present both amyloid plaques and NFT. Each point represent one iNPH patient

Due to the fact that iNPH and AD can coexist histopathological analysis was performed, and brain biopsies divided into three groups according to the presence or absence of amyloid plaques and NFT (Aβ^−^/tau^−^, Aβ^+^/tau^−^, Aβ^+^/tau^+^) (Material and methods, Table 1 and Additional file 1: Figure S1). The length of MERCS was significantly shorter in the Aβ^+^/tau^+^ group as compared to the Aβ^+^/tau^−^ and Aβ^−^/tau^−^ groups (Fig. 4b and Additional file 1: Table S1). These data were further corroborated analysing the median of MERCS length of Aβ^+^/tau^+^ samples (median = 118.5) compared to Aβ^−^/tau^−^ (median = 162.1) and Aβ^+^/tau^−^ (median = 151.1) samples. Meanwhile, the number of MERCS was similar between the three groups (Fig. 4a and Additional file 1: Table S1). Quantifications of the number of mitochondrial profiles and mitochondrial perimeter revealed no significant differences between the three different groups (Additional file 1: Figure S4a, b and Table S1).

In a previous study by Area-Gomez et al. MERCS length was categorized into three groups: punctate (50 nm), long (50-200 nm) and very long (> 200 nm) [1]. We decided to group our data accordingly to assess the distribution of the contacts length analysed. We observed that while the Aβ^−^/tau^−^ group present a higher percentage of very long contacts (40.5%) when compared to punctate contacts (26.9%), the Aβ^+^/tau^+^ group showed the opposite pattern with less very long contacts (22.7%) and more punctate contacts (50.3%) (Fig. 4c). These findings support the idea that the average length of MERCS is shorter in patients’ samples with amyloid plaques and NFT.

## Discussion

In this study, we have characterized for the first time membrane contact sites in human brain biopsies. We show several electron micrographs from TEM of a variety of membrane contact sites, including mitochondria-PM, mitochondria-nucleus, mitochondria-Golgi and mitochondria-lysosome contacts. Due to the method used to collect the samples and due to the ultrastructural similarities between different cell types it was not possible to identify neither which cortical layer nor the type of cell analysed. Nevertheless, our data show the existence of previously described membrane contact sites in human brain cells. Our study expands on the extensive work carried out by Wu and colleagues in which they assessed MERCS distribution in mouse brain tissue. Importantly, we similarly showed the existence of MERCS in intact pre- and post-synaptic terminals in this material, hence opening up potential avenues for research on the role of these structures in these important areas of neurons and glial cells [38].

Even though MERCS have been shown to be dysregulated in a variety of neurodegenerative diseases, the mechanisms behind this disruption and the role of MERCS in different pathologies is still largely unknown. Here, we used iNPH patient samples to assess the relationship between MERCS structure and different clinical parameters of these patients. Interestingly, iNPH patients with comorbities with AD, VaD/AD or LBD/VaD showed an increased number of MERCS per cell profile when compared to non-demented patients. In line with these data low MMSE scores correlated with increased numbers of MERCS per cell profile. Curiously, the number and function of MERCS have been reported to be increased in e.g. AD and to be decreased in e.g. frontal temporal dementia, showing the dubious dysregulation of these contacts in different diseases [7]. More biopsies of patients diagnosed with dementia would be necessary to assert whether these results represent a population’s trend or just an artefact of our small sample set. Nevertheless, to our knowledge this is the first time that a connection between late stages of dementia and an increased number of MERCS per cell profile has been observed and reported in human brain tissue.

The major risk factor for neurodegenerative diseases is ageing. Neurons are non-dividing post-mitotic cells and are particularly affected by noxious stimuli. Ageing neurons experience increased oxidative stress, accumulation of damaged proteins and energy imbalance. Due to their substantial energetic demands and delicate physiology, neurons are more sensitive to cell stress leading to deregulated homeostasis and death [21]. Interestingly, we observed a positive correlation between age of the patients analysed and the number of MERCS per cell profile. Although mitochondria are commonly associated with ATP production they also have a major role in controlling cell death processes. Both apoptosis and necrosis can be triggered by changes in Ca^2+^ levels in mitochondria. Influx of Ca^2+^ has been shown to induce opening of the mitochondrial permeability transition pore which leads to loss of the mitochondrial membrane potential leading to cell death. Ca^2+^ has also been described as a regulator of several mitochondrial dehydrogenases in the Krebs cycle including pyruvate dehydrogenase. In fact, increased levels of Ca^2+^ in mitochondria lead to phosphorylation of pyruvate dehydrogenase reducing its activity and affecting ATP levels. Since it has been shown that increased connectivity between ER and mitochondria leads to increased shuttling of Ca^2+^ from ER to mitochondria these changes could lead to neuronal death. [2, 7]. Therefore, we believe that the reported increase of MERCS per cell profile, both in demented patients and with increasing age, could contribute to synaptic loss and cognitive decline.

Recently, we and others have shown in different models that Aβ causes an increased connectivity between mitochondria and ER [13, 27, 39]. We have also observed that Aβ is formed in subcellular fractions enriched in MAM and MERCS modulation leads to changes in intra- and extracellular Aβ levels [18, 28]. However, most of these studies have relied on in vitro experiments and mouse models with increased levels of Aβ which do not always mimic perfectly the progression of the pathology as observed in humans. In concordance, we report a positive correlation between ventricular Aβ42 levels and number of MERCS in this study; indicating an increased connectivity between the two organelles. However, we should consider the limitation of this correlation since we are comparing ventricular CSF Aβ42 levels and MERCS number obtained from analysed organelles within the cell. Aβ has been shown to be cleared both inside and outside the cell. Inside the cell Aβ can be degraded by insulin degrading enzyme in the cytosol and endosomes, and presequence peptidase in the mitochondria [20]. Aβ can also be cleared extracellularly by different mechanisms: cleavage by neprilysin (on the cell membrane), transport across the blood-brain-barrier, drainage into CSF via interstitial fluid bulk flow or by absorption of CSF into the lymphatic and circulatory system [34]. Furthermore, it is thought that while Aβ40 is mainly degraded intracellularly, Aβ42 is degraded extracellularly [11]. Surprisingly, no correlation between lumbar CSF Aβ42 and MERCS were found. Likewise, no significant correlations were observed between CSF total-tau or CSF pTau and the number of MERCS per cell profile. Up to date only two publications have reported a connection between tau protein and MERCS. Perreault and colleagues showed that the tau mutant JNLP3 increased the proximity between mitochondria and ER [23], and Cieri and colleagues showed that a form of truncated tau (caspase 3-cleaved 2N4RΔC20 tau) has the same effect [3]. Yet, the role of pathological pTau on ER-mitochondria dynamics remains largely unknown. So far, our data suggests that increased levels of ventricular Aβ and number of MERCS are positively correlated, unlike tau protein and MERCS. Further studies will be required to confirm our findings and investigate further the role of Aβ and tau on MERCS dynamics.

As already mentioned iNPH patients often show comorbidities with AD, including the respective hallmarks, amyloid plaques and NFT. Even though some studies have shown that Aβ affects MERCS no reports have revealed the effect of amyloid plaques and NFT in MERCS. Therefore, the relationship between these hallmarks and MERCS in human brain remains elusive. We decided to categorize our samples according to the presence or absence of amyloid plaques and NFT and investigate if amyloid plaques alone or together with NFT had an impact on MERCS. Our data show that patients with both amyloid plaques and NFT presented shorter MERCS as compared with patients lacking these hallmarks or presenting just amyloid plaques (Fig. 4b and c). No changes in the number of MERCS per cell profile were detected here. At a first glance, these findings may seem to be contradictory to the previous correlation results discussed above: simultaneous presence of amyloid plaques and NFT cause decreased connectivity between mitochondria and ER, while Aβ (monomeric/oligomeric) cause increased contact between the two organelles. However, here samples were grouped based on histological characterization of amyloid plaques and NFT staining while soluble Aβ-levels were not considered. Importantly, several studies show that oligomeric species of Aβ, and not plaques per se, are the main driver of toxicity in AD. In fact, there is a lack of correlation between the plaque burden and the progression of AD [9, 19]. Furthermore, there is substantial neuronal death in regions lacking plaques, while plaques were found in patients with no cognitive impairment [30, 32]. Our present data suggests that in samples with amyloid plaques alone (NFT negative samples) MERCS are not affected, while the levels of ventricular CSF Aβ42 correlate with the number of these contacts. Therefore, we postulate that intracellular Aβ and amyloid plaques seem to have different effects on MERCS, however further studies are required to elucidate the underlying mechanisms.

## Conclusions

In summary, we show that iNPH patients diagnosed with either AD, VaD or LBD present an increased number of MERCS per cell profile. We also show that the number of MERCS positively correlates with age and levels of ventricular CSF Aβ42. In addition, the length of MERCS was decreased in iNPH patients presenting both amyloid plaques and NFT. Together, these findings strengthen the hypothesis that MERCS affect cell homeostasis and could be one of the players in the neurodegenerative process found in different diseases like AD and iNPH. Future studies in relevant models are needed to reveal the exact cellular mechanisms and can also be used to test to drug candidates correcting the ER-mitochondria interplay.

## Additional file

Additional file 1: **Figure S1.** Immuno-labelling of biopsies of frontal cortices of iNPH patients. Representative immunohistochemistry pictures of frontal cortices of patients analysed. Patients were divided in groups according to the presence or absence of amyloid plaques and NFT. Anti-Aβ antibody (6F/3D, M0872; Dako) (first column) and anti-p-Tau antibody (AT8) (second column) were used. The arrow indicate a NFT and the star indicates neuropil threads. Scale bar = 500 μm. **Table S1.** Electron microscopy measurements and respective averages. **Figure S2.** Mitochondria number and perimeter are not significantly changed in patients diagnosed with dementia. Quantification of **a** number and **b** perimeter of mitochondria profiles from the electron micrographs of iNPH patients’ biopsies according to dementia diagnose. Non-demented patients are #1 to #4 and #6 to #12, demented patients are #5, #13 and #14. Each point represent one iNPH patient. Quantification of **c** number and **d** perimeter of mitochondria profiles from the electron micrographs of iNPH patients’ biopsies according to MMSE. MMSE scores represent: MMSE ≥27 – No significant cognitive impairment, 23 ≤ MMSE ≤26 – Minor cognitive impairment, MMSE ≤22 – moderate or severe cognitive impairment. **Figure S3.** Number and length of MERCS negatively with MMSE. Representation of the correlation between MMSE and mitochondria-ER contact sites **a** number and **b** length. Linear regression was performed and the Pearson correlation coefficient (r) calculated. Each point represent one iNPH patient. **Figure S4.** Amyloid plaques and NFT have no effect in the number of mitochondria profile nor mitochondria perimeter. Quantification of **a** number and **b** perimeter of mitochondria profiles from the electron micrographs of iNPH patients biopsies. Aβ^−^/tau^−^ patients present no amyloid plaques nor NFT; Aβ^+^/tau^−^ patients presents amyloid plaques but not NFT; and Aβ^+^/tau^+^ patients present both amyloid plaques and NFT. Each point represent a different iNPH patient. (PDF 16959 kb)
