# Supplementary material for: Alterations in mitochondria-endoplasmic reticulum connectivity in human brain biopsies from idiopathic normal pressure hydrocephalus patients
Source: Acta Neuropathol Commun. 2018 Oct 1;6:102. doi: 10.1186/s40478-018-0605-2 (PMC6166280; doi:10.1186/s40478-018-0605-2)
Supplement: Supplementary file 1 — Figure S1. Immuno-labelling of biopsies of frontal cortices of iNPH patients. Representative immunohistochemistry pictures of frontal cortices of patients analysed. Patients were divided in groups according to the presence or absence of amyloid plaques and NFT. Anti-Aβ antibody (6F/3D, M0872; Dako) (first column) and anti-p-Tau antibody (AT8) (second column) were used. The arrow indicate a NFT and the star indicates neuropil threads. Scale bar = 500 μm. Table S1. Electron microscopy measurements and respective averages. Figure S2. Mitochondria number and perimeter are not significantly changed in patients diagnosed with dementia. Quantification of a number and b perimeter of mitochondria profiles from the electron micrographs of iNPH patients’ biopsies according to dementia diagnose. Non-demented patients are #1 to #4 and #6 to #12, demented patients are #5, #13 and #14. Each point represent one iNPH patient. Quantification of c number and d perimeter of mitochondria profiles from the electron micrographs of iNPH patients’ biopsies according to MMSE. MMSE scores represent: MMSE ≥27 – No significant cognitive impairment, 23 ≤ MMSE ≤26 – Minor cognitive impairment, MMSE ≤22 – moderate or severe cognitive impairment. Figure S3. Number and length of MERCS negatively with MMSE. Representation of the correlation between MMSE and mitochondria-ER contact sites a number and b length. Linear regression was performed and the Pearson correlation coefficient (r) calculated. Each point represent one iNPH patient. Figure S4. Amyloid plaques and NFT have no effect in the number of mitochondria profile nor mitochondria perimeter. Quantification of a number and b perimeter of mitochondria profiles from the electron micrographs of iNPH patients biopsies. Aβ−/tau− patients present no amyloid plaques nor NFT; Aβ+/tau− patients presents amyloid plaques but not NFT; and Aβ+/tau+ patients present both amyloid plaques and NFT. Each point represent a different iNPH patient. (PDF 16959 kb) [file 40478_2018_605_MOESM1_ESM.pdf]

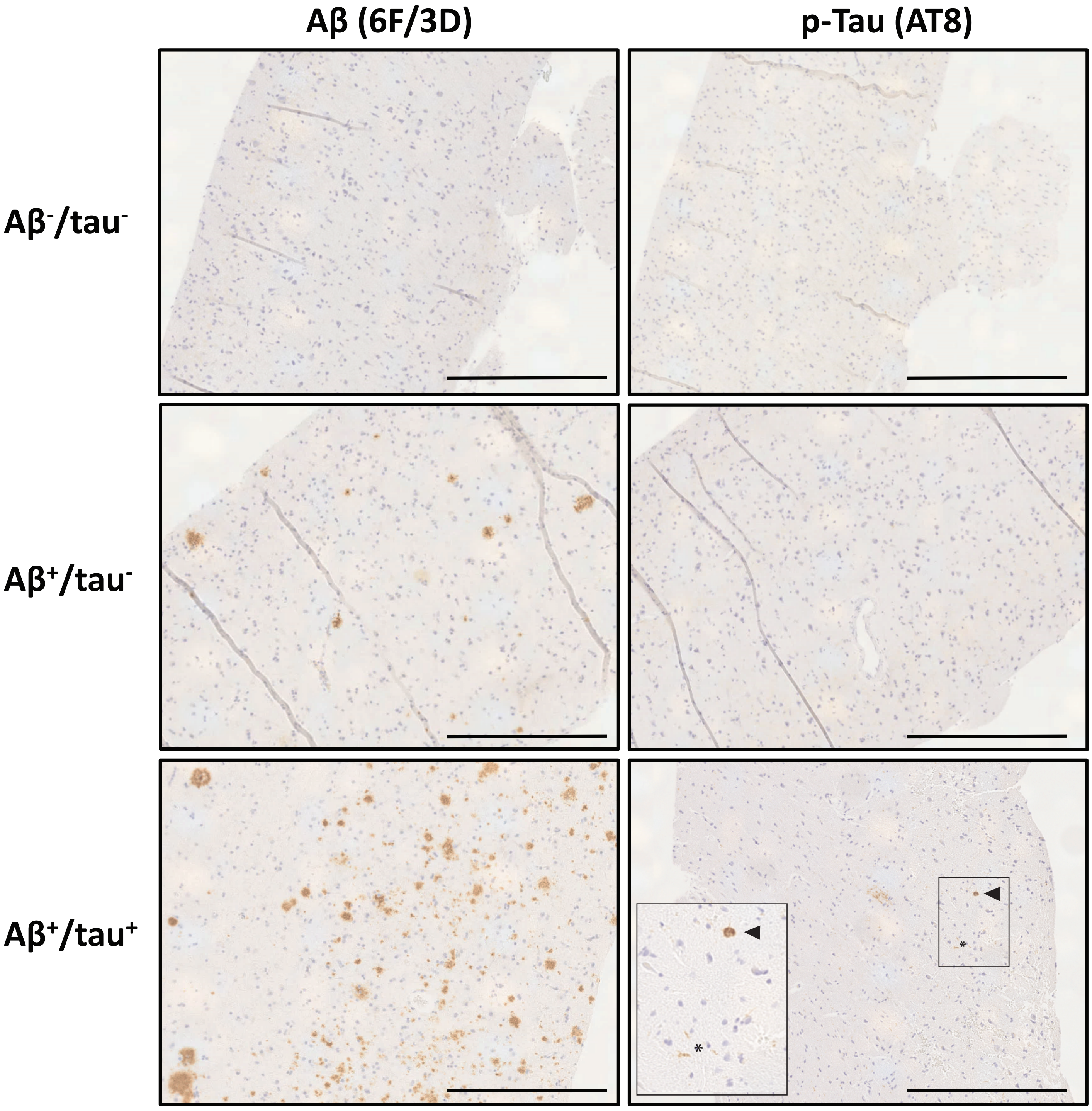
Aβ (6F/3D)
p-Tau (AT8)
Aβ-/tau-
Aβ+/tau-
Aβ+/tau+

Table S1. Electron microscopy measurements of MERCS and mitochondrial profile and respective averages.

	#	Number of MERCS	Number of Mitochondria profiles	MERCS length /nm	Mitochondria perimeter/nm
$A\beta^-/tau^-$	1	10,70	22,00	197,11	1246,93
	2	5,86	22,88	138,01	1260,17
	3	4,20	7,90	147,52	1177,79
	4	3,63	5,73	188,16	1275,76
$A\beta^+/tau^-$	5	13,50	26,40	170,36	1105,94
	6	8,50	19,80	207,08	1394,96
	7	3,30	9,10	180,08	1250,19
	8	3,00	6,50	163,18	1330,15
	9	3,70	12,00	199,39	1646,22
$A\beta^+/tau^+$	10	5,25	12,78	119,15	1132,40
	11	5,90	8,00	134,29	1204,85
	12	5,50	11,00	129,06	1316,41
	13	6,17	20,86	176,21	1309,95
	14	8,10	16,10	133,37	1219,76
**Average values**					
$A\beta^-/tau^-$		6.10 ± 1.60	167.70 ± 14.64	14.63 ± 4.53	1240.16 ± 21.61
$A\beta^+/tau^-$		6.4 ± 2.04	184.02 ± 8.38	14.76 ± 3.67	1345.49 ± 89.33
$A\beta^+/tau^+$		6.18 ± 0.50	138.42 ± 9.82	13.75 ± 2.21	1236.68 ± 34.57

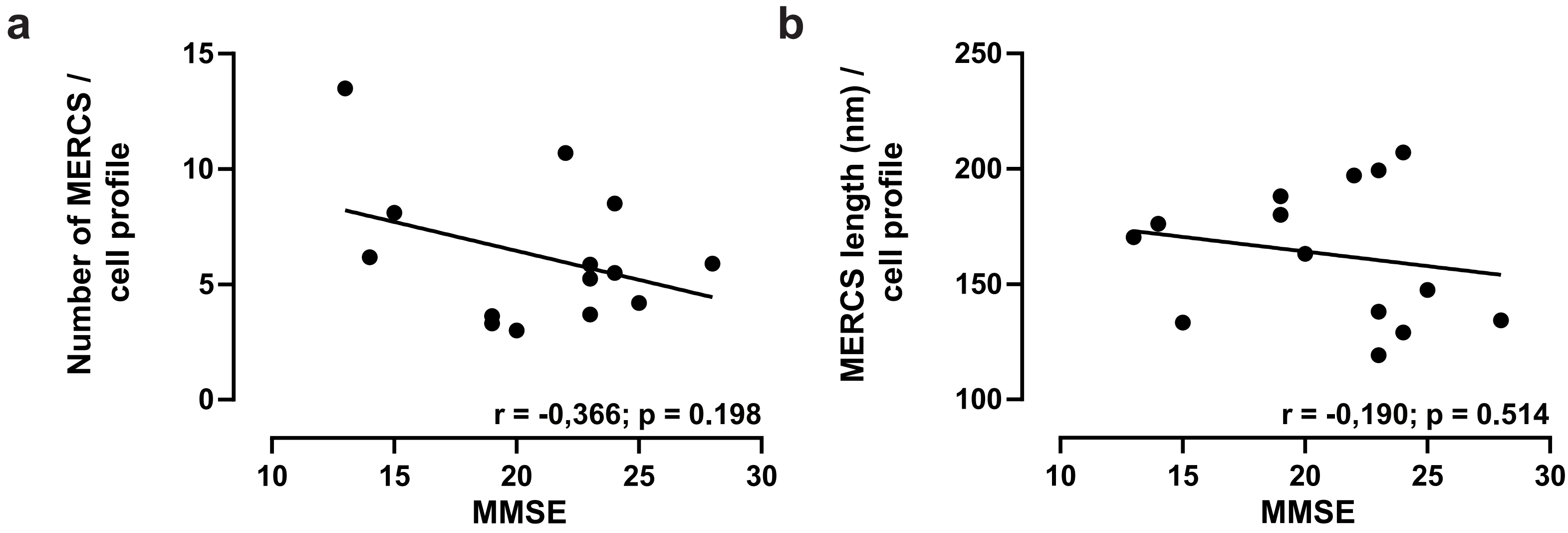
a
b
Number of MERCS / cell profile
15
10
5
0
r = -0,366; p = 0.198
10
15
20
25
30
MMSE
MERCS length (nm) / cell profile
250
200
150
100
r = -0,190; p = 0.514
10
15
20
25
30
MMSE

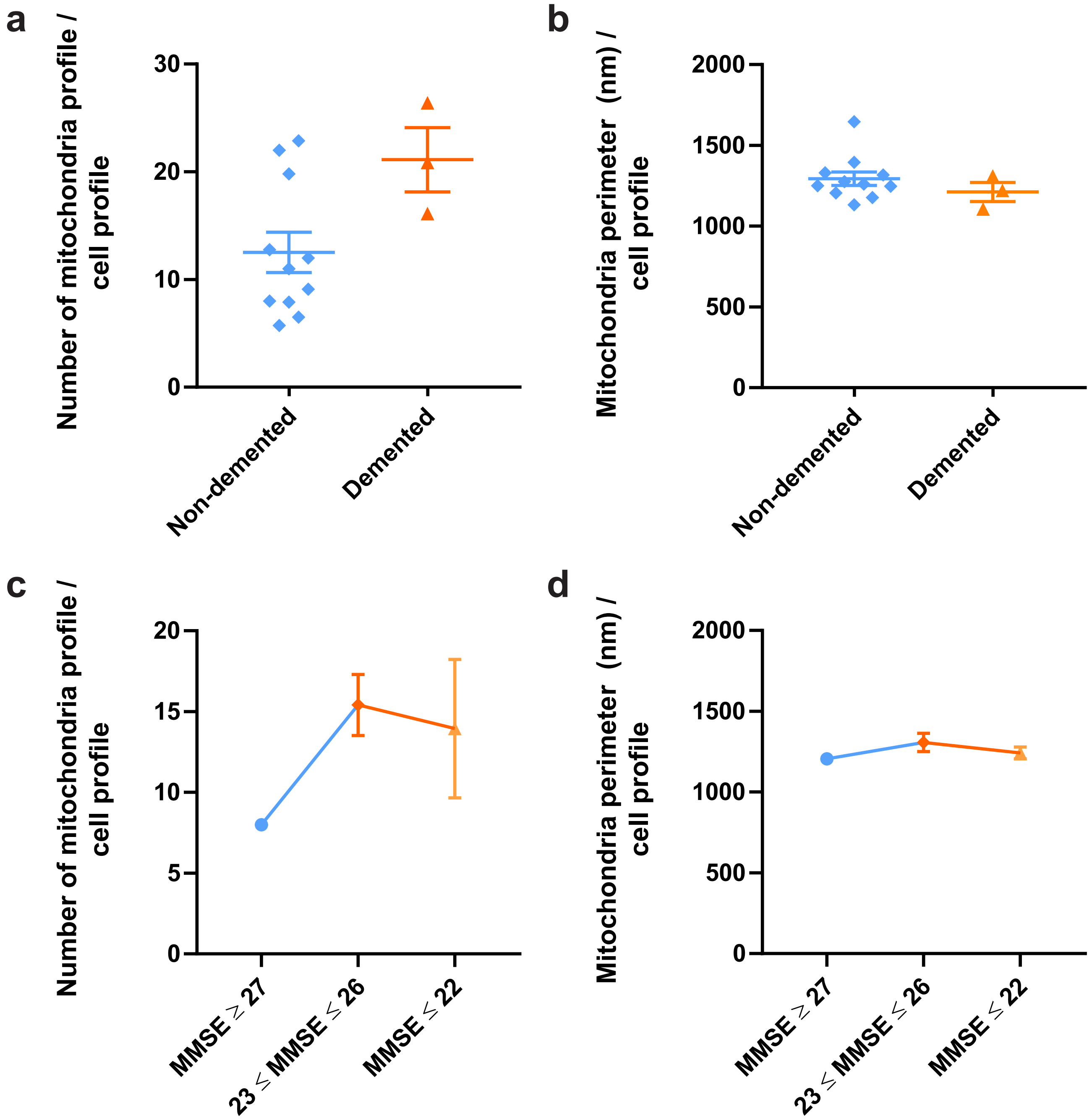

a
Number of mitochondria profile / cell profile
30
20
10
0
Non-demented
Demented
b
Mitochondria perimeter (nm) / cell profile
2000
1500
1000
500
0
Non-demented
Demented
c
Number of mitochondria profile / cell profile
20
15
10
5
0
MMSE ≥ 27
23 ≤ MMSE ≤ 26
MMSE ≤ 22
d
Mitochondria perimeter (nm) / cell profile
2000
1500
1000
500
0
MMSE ≥ 27
23 ≤ MMSE ≤ 26
MMSE ≤ 22

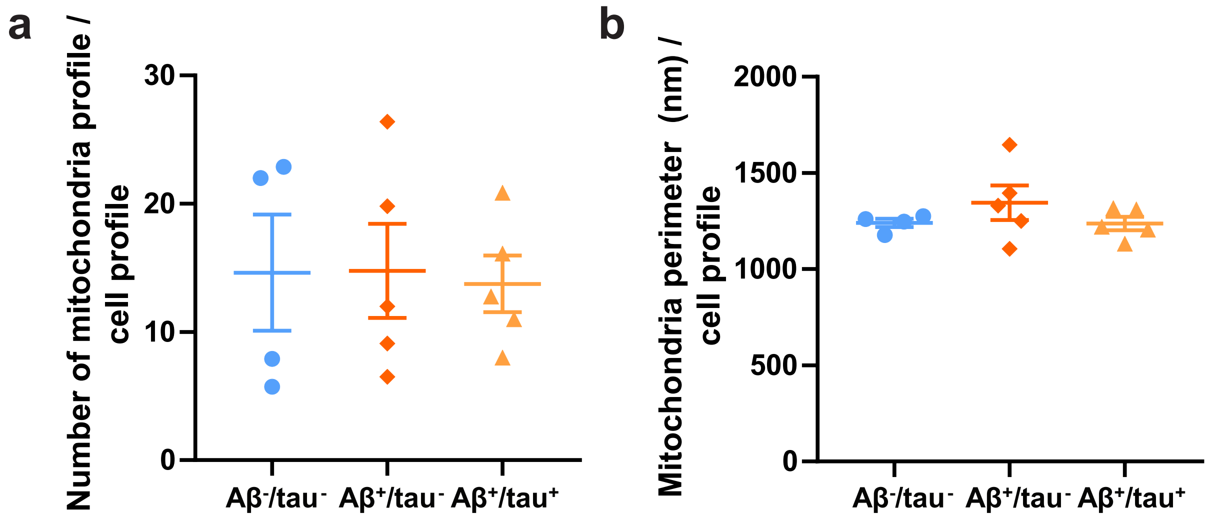

a
Number of mitochondria profile / cell profile
0
10
20
30
Aβ-/tau-
Aβ+/tau-
Aβ+/tau+
b
Mitochondria perimeter (nm) / cell profile
0
500
1000
1500
2000
Aβ-/tau-
Aβ+/tau-
Aβ+/tau+
